# Capturing coacervate formation and protein partition by molecular dynamics simulation†

**DOI:** 10.1039/d2sc01164f

**Published:** 2022-12-24

**Authors:** Yang Liu, Xinyan Wang, Zhili Wan, To Ngai, Ying-Lung Steve Tse

**Affiliations:** a College of Polymer Science and Engineering, State Key Laboratory of Polymer Materials Engineering, Sichuan University Chengdu 610065 China; b Department of Chemistry, The Chinese University of Hong Kong Sha Tin Hong Kong China tongai@cuhk.edu.hk stevetse@cuhk.edu.hk; c School of Food Science and Engineering, South China University of Technology Guangzhou China

## Abstract

Biomolecules localize and function in microenvironments where their local concentration, spatial organization, and biochemical reactivity are regulated. To compartmentalize and control the local properties of the native microenvironment, cellular mimics and artificial bioreactors have been developed to study the properties of membraneless organelles or mimic the bio-environment for life origin. Here, we carried out molecular dynamics simulation with the Martini 3.0 model to reproduce the experimental salt concentration and pH dependency of different complex coacervates. We showed that coacervates inside vesicles are able to change their shape. In addition, we used these coacervate systems to explore the partitioning of the ubiquitous cytoskeletal protein actin and found that actin spontaneously partitions to all the coacervate peripheries. Therefore, we believe that our study can provide a better understanding of the versatile coacervate platform, where biomolecules partition and gather to fulfill their biological duties.

## Abstract 2

We carried out molecular dynamics simulation with the Martini 3.0 model to reproduce the experimental salt concentration and pH dependency of different complex coacervates. We showed that coacervates inside vesicles are able to change their shape.

## Graphical abstract

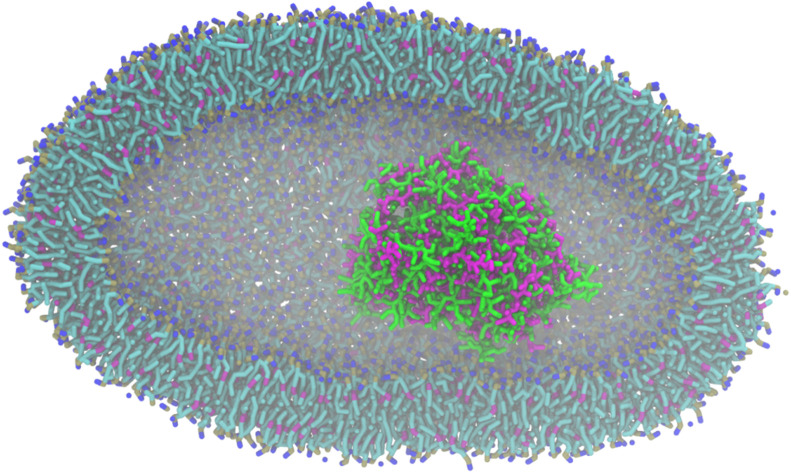

## Introduction

Even though science has been advancing for centuries, how life emerged from non-living chemicals more than 3.5 billion years ago on Earth remains poorly understood. The RNA hypothesis^3^ assumes that RNA plays a key role in the origin of life, which arose before the evolution of DNA and proteins. However, this assumption is challenging for RNA passive encapsulation by membrane-like amphiphile self-assemblies such as vesicles especially in the absence of binding interactions.^4,5^ An alternative compartmentation scheme becomes possible if RNA is contained in a membraneless microdroplet, providing a liquid “proto-cytoplasm” environment.^6^ The coacervate droplets are spontaneously assembled through the physicochemical phenomenon of liquid–liquid phase separation, forming dense polymer-rich coacervates and an external homogenous milieu.^7^ Proto-biomolecules, such as RNA, can be accumulated within coacervate to several orders of magnitude higher in concentration than the surrounding environment.^8^ Besides, the membraneless characteristic of coacervate enables it to easily take up and dynamically exchange biomolecules from the surroundings and neighboring coacervates. In addition, the associative interactions between oppositely charged oligomeric or polymeric molecules and their diverse side group functionalities allow selectivity for guest molecules.^9^ Therefore, the coacervate droplet is a promising candidate for a protocell and may have played an important role in the original life.

Coacervate droplets also play an important role in modern cells. Due to the complexity of the native cytoplasm and the cellular microenvironments, the development of cellular mimics to compartmentalize and regulate the local physicochemical properties has drawn great attention lately.^10–13^ In modern cells, membraneless organelles, such as a P-body, Cajal body, stress granule, are some of the completed condensed bodies containing various intrinsically disordered proteins, ATP/ADP, and nucleic acids.^14,15^ They are mainly responsible for nucleic acid processing and regulation and enhancing catalysis. However, many aspects related to the formation of membraneless organelles, aging, and their influence on cellular processes^16,17^ are far from understood.^18^ Experimentalists use liquid-like coacervate models, usually made of oppositely charged synthetic polymers and biomolecules, to mimic and precisely control the properties, behavior, and functions of membraneless organelles.^16,19^ Comprehending the mechanism of how microenvironment properties fine-tune protein partitioning and activity could lead to a better understanding of membraneless organelles as well as design principles for synthetic biology and engineering applications.^20,21^ Therefore, understanding polyelectrolyte coacervate is crucial to exploring the origin of life^22,23^ and the properties of membraneless organelles.^10,24^

The formation of polyelectrolyte coacervate starts from electrostatic attractions between oppositely charged polyelectrolytes, followed by reconstructing water and releasing counterions.^25^ This formation process depends on charge-driven factors, such as ionic strength, pH, and salt density.^26–28^ There remains plenty of water within the fluid coacervate regions, which display low surface tension with water^29^ and are viscoelastic.^25,30^

Molecular dynamics (MD) simulation has emerged as a unique and indispensable tool to complement conventional experimental approaches. It is able to describe target systems at the molecular level and act like a “computational microscope”. A Martini 3.0 force field^2^ has been chosen in this study because the work by Tsanai *et al.*^31^ has shown that Martini is able to reproduce salt-dependent coacervation and the partitioning of nucleotides between the coacervate and water region at near-atomistic resolutions. In our MD simulations, we explored the formation of self-assembling polypeptide coacervates and the partitioning of the ubiquitous cytoskeletal protein actin to the coacervate aggregate. In addition to the influence of salt studied by Tsanai *et al.*,^31^ we found that the Martini 3.0 force field can also reproduce the influence of pH on coacervate formation. Furthermore, we observed that the actin partitions into liquid polymer-rich droplets and preferably localizes at the droplet periphery. We believe that our research can provide more insight into the mechanism of liquid–liquid phase separation and lead to a better understanding of coacervate partitioning regulation.

## Results and discussion

### Salt-dependent coacervation

**Fig. 1:**
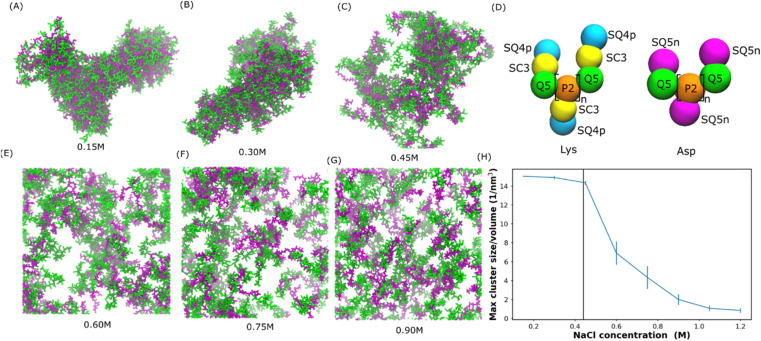
Salt-dependent Lys/Asp coacervate. (A–C and E–G) Snapshots of the final state of 100 pairs of Lys and Asp polymer systems at different salt concentrations. Lys and Asp are shown in green and purple, respectively. Water and ions are not shown for clarity. (D) Martini models of poly Lys and Asp peptides. (H) Maximum peptide cluster size normalized by system volume as a function of NaCl concentration. The vertical line represents the experimental transition salt concentration.^1^

Mediated by charge screening, coacervate could dissolve upon increasing the salt concentration.^1^ In order to study the salt concentration effect on the complexation of polyelectrolytes, we set up simulations with randomly distributed 30-mer of aspartic acid (Asp) and 30-mer of lysine (Lys) polymers using the Martini 3.0 force field.^2^ The computational scheme is similar to that in ref. 31. The secondary structure was decided based on an all-atom simulation as shown in Fig. S1.† Both polypeptides were adapted to the l-homochiral structure, which is the most common stereoisomer in nature. Coil was the predominant secondary structure for both peptides in our all-atom simulations (Fig. S1†). In the Martini force field, the secondary structure is considered as coil except when more than three non-coil residues with the same secondary structures are consecutively connected. Therefore, a coil secondary structure backbone was chosen for both Asp and Lys polypeptides in our simulations. The polypeptides were uniformly distributed at various sodium chloride (NaCl) concentrations from 0.15 to 0.9 M. Snapshots at the ends of the simulations are shown in Fig. 1(A–C and E–G). We can see that coacervate is formed at 0.15 M NaCl, while gradually becoming more soluble as the salt concentration increases. At 0.3 M NaCl, coacervate is stable while starting to dissolve at 0.45 M. Beyond 0.6 M NaCl, the coacervates are fully dissolved. To quantify the salt concentration at which coacervation is observed in our simulations, we calculated the radial distribution functions (RDFs), contact number between peptides and solvent molecules, and the maximum peptide cluster size as shown in Fig. S4† and 1H. As shown in Fig. S4,† the location of the first valley of RDF (0.6 nm) was used as a cut-off for the computations of the contact number and cluster size, in which the cut-off was the largest distance to be considered as contact or within a cluster. As shown in Fig. S4,† the contact number between Lys and Asp decreases with the increase in the salt concentration, while the contact number between polypeptides and water or ions increases with the salt concentration. In addition, the maximum cluster size decreases as the salt concentration increases as shown in Fig. 1H. These results agree with the observations in Fig. 1(A–C and E–G) and are controlled by the screening effect of NaCl. The maximum cluster size plot in Fig. 1H shows a sudden decrease at NaCl concentrations between 0.45 and 0.6 M, suggesting a transition from the coacervation to non-coacervation phase. This transition concentration range agrees with the snapshots in Fig. 1C and E. In the experiment,^1^ for a 1 : 1 Lys–Asp polymer mixture, coacervation occurs at 0.44 ± 0.01 M KCl. This value is close to the range observed in our simulation. We also investigated the salt concentration effect on a coacervate composed of poly-l-lysine and poly-(d, l)-glutamic acid with a coil secondary structure (coil Lys/Glu) (also see Tsanai *et al.*^31^ for a similar scheme). The transition happens between 0.4 M and 0.55 M NaCl, which agrees with the experimental value at about 0.4 M.^32^ Therefore, we believe that our models are able to, at least semi-quantitatively, capture the experimentally observed salt-dependence of coacervation. Details of the salt concentration effect on Glu/Lys coacervate are included in the ESI.†

### pH-Dependent coacervation in a lipid vesicle

Understanding membrane-confined liquid–liquid phase separation is an important step toward artificial organelles.^33^ To control the coacervate droplets within lipid vesicles, pH responsiveness of a polycation above and below its p*K*_a_ was used to drive the liquid–liquid phase separation. It was found in the experiment^34^ that as pH increases above the p*K*_a_ of Lys, the dissolution of the coacervate droplets is triggered, and when the pH decreases below the p*K*_a_, the coacervate droplets re-emerge. Apart from coil Lys/Asp and coil Lys/Glu systems, coacervates composed of poly-l-lysine and poly-l-glutamic acid multi-peptides with a β-sheet secondary structure (β sheet Lys/Glu), which was studied in ref. 31, were also considered here.

**Fig. 2:**
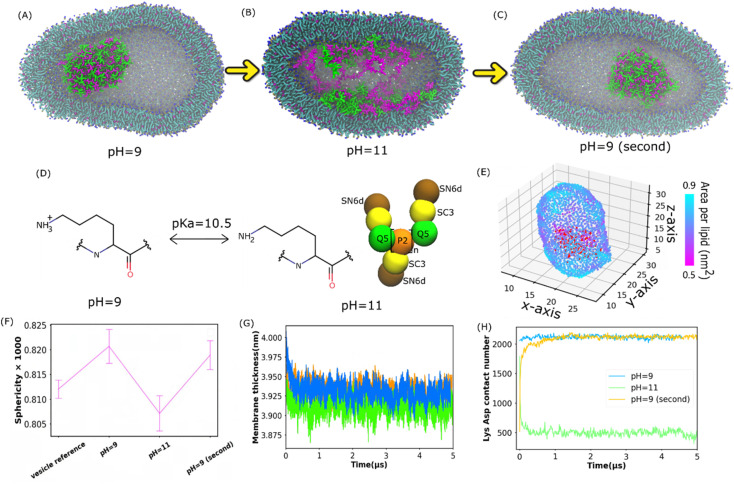
Reversible formation of Lys/Asp coacervates in POPC vesicles by changing pH. (A–C) illustrate the final states of the Lys/Asp polypeptide systems at different pH values. The last frames in (A) and (B) were used as the initial configurations of the simulations in (B) and (C), respectively. (D) Chemical structures of Lys switching between a cationic polymer and an uncharged polymer (p*K*_a_ = 10.5). The bead types were chosen based on the Martini 3.0 protocol.^2^ (E) Area per lipid of the inner leaflet of the vesicle (F) sphericity of different systems. A POPC vesicle without multi-peptides is the vesicle reference. (G and H) Membrane thickness and Lys/Asp contact number as a function of time at different pH values. (G) and (H) Share the same legend.

To see if the reversible process of liquid–liquid phase separation can be reproduced in our simulations, we applied the Martini 3.0 model as shown in Fig. 2(A–C). The Lys/Asp coacervate droplet was encompassed in the 1-palmitoyl-2-oleoyl-*sn*-glycero-3-phosphocholine (POPC) vesicle. The amine group of Lys was deprotonated and the Lys molecule was overall neutral when the pH was higher than the Lys p*K*_a_ (Fig. 2D). The Martini models at pH = 9 were the same as those in Fig. 1D, and the deprotonated Lys model at pH = 11 is illustrated in Fig. 2D. As shown in Fig. 2A, at pH = 9, a coacervate droplet was formed in the vesicle. The Lys/Asp contact number was ∼2000 and stable throughout the simulation, as shown in Fig. 2H. The peptides/POPC contact number is very low (Fig. S6†). Using the last frame of the simulation shown in Fig. 2A as a starting structure, we performed another simulation with the deprotonated Lys topology at pH = 11 as illustrated in Fig. 2D, and the coacervate droplet dissolved (Fig. 2B). The Lys/Asp contact number decreased to ∼500 within 1 μs and stayed ∼500 for the rest of the simulation (Fig. 2H). The peptides/POPC contact number increased and reached an equilibrium value within 1 μs (Fig. S6†). Using the dissolved configuration (Fig. 2B), we carried out a third simulation at pH = 9 with the Lys topology shown in Fig. 1D. The coacervate recovered in the vesicle. The Lys/Asp contact number increased back to ∼2000 and the peptides/POPC contact number returned to the lower value within 1.5 μs (Fig. S6†). Similar reversible processes also occur for coil Lys/Glu and β sheet Lys/Glu coacervate as shown in Fig. S7† and the corresponding analysis is shown in Fig. S5 and S6.† These reversible processes in our simulations agree with the experimental observations.^34^ It is well known that coacervate is mainly stabilized by the attraction between opposite charges of polymers.^18^ At pH = 11, because Lys polypeptides were neutral, the electrostatic interactions between the coacervate components were annulled. Therefore, the coacervate dissolved at pH = 11. On the other hand, coacervate was recovered at pH = 9 because of the restored electrostatic interactions.

Apart from the formation and dissolution of the coacervate droplet, we also observed that the shapes of the vesicles can be affected by the presence of charged polypeptides. Sphericity (see the Methods section) was computed as shown in Fig. 2F, S8 and S15A.† The pure POPC vesicle (without multi-peptides) adapted an elliptical shape, agreeing with the results in ref. 35. Adding multi-peptides into the vesicle increased the sphericity at pH = 9, but decreased it at pH = 11 (Fig. 2F). Usually, a vesicle shape transformation arises from either an osmotic imbalance or a change in membrane curvature, which could be caused by the polymers interacting with the lipids. We found that the higher NaCl osmotic imbalance increases the sphericity as shown in Fig. S15A (see the ESI† for more). Adding multi-peptides into the vesicle also increases the osmotic imbalance. As shown in Fig. 2E and S11,† there was no local area per lipid larger than 0.9 nm^2^. Thus, no water pores were formed on the vesicle surfaces. At pH = 9, peptides barely came into contact with the vesicle membrane as shown in Fig. 2H and S6.† Thus, the increased sphericity was probably caused mainly by osmotic imbalance. At pH = 11, many peptide chains attached to the inner surfaces of the vesicle (Fig. 2H and S6†), increased the area per lipid (Fig. 2E, S10 and S11†), and decreased the membrane thickness (Fig. 2G and S9†) and mean order parameter (Fig. S12†). It was reported that when the coacervates attach to the membrane surface in the Martini 3.0 model, they could induce a strong curvature in the membrane.^36^ A similar effect can be found in all of our vesicle simulations, and the sphericity was found to be lower at pH = 11. However, an aspherical vesicle shape was not observed in the experiments.^36,37^ This was probably due to the different size ratios of the vesicle and the coacervate in the simulation and the experiments.^36,37^

### Partitioning of actin protein

**Fig. 3:**
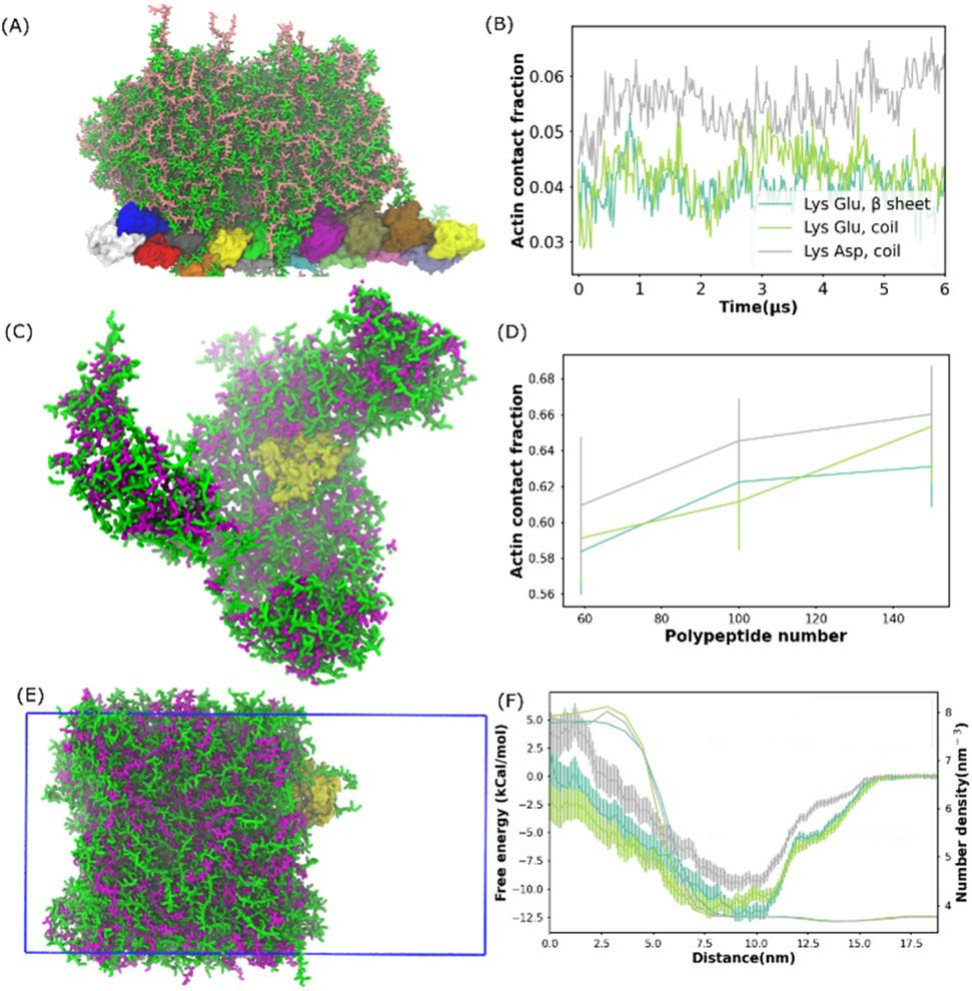
Actin protein partition in polypeptide coacervate. (A) Snapshot of the final state for a system composed of one actin filament and 314 pairs of coil Lys/Glu polypeptides. (B) Contact fraction between the actin filament and coacervate as a function of time for systems shown in (A) and S6.† (C) Snapshot of the final state for a self-assembled system composed of G-actin and 100 pairs of coil Lys/Asp poly-peptides. (D) Actin contact fraction as a function of the number of polypeptide pairs for the self-assembled systems. (E) Snapshot of G-actin attached to the coacervate interface. This configuration was used to compute the free energy profile of actin partitioning into the coacervate phase. The simulation box is shown as the blue frame. (F) Free energy profile and coacervate number density as a function of the distance from 0 nm to 18 nm. (B), (D) and (F) Share the same legend.

The ubiquitous cytoskeletal protein actin was found to affix to the periphery of the liquid coacervate droplet.^38^ However, the physical mechanism for the peripheral localization of actin filaments (F-actin) in coacervate droplets has not been fully understood. To better understand this process, we performed additional MD simulations of a well phase-separated polypeptide coacervate in the presence of an F-actin. Coacervates were composed of 314 pairs of coil Lys/Glu, β sheet Lys/Glu and coil Lys/Asp multi-peptides. In all the cases, F-actin attached to the surfaces of the coacervates and this attachment lasted for more than 5 microseconds as shown in the last frame of the simulation in Fig. 3A and S14.† This observation is consistent with the experimental results,^38^ in which the polypeptides composed of poly-l-lysine and the polyanion poly-(l, d)-glutamic acid correspond to coil Lys/Glu in our simulations. To quantify the interaction between the actin filament and the coacervate, we computed the actin contact number as shown in Fig. 3B. After the contact fractions (defined in eqn (1)) plateaued, the actin contact of the coil Lys/Asp system was higher than those of the coil Lys/Glu and β sheet Lys/Glu systems, which were similar. A magnesium CG bead was embedded in each actin monomer. Thus, the locations of the magnesium ions could be used to map the shape of the F-actin. The presence of coacervate could induce a slight bending of F-actin as shown in Fig. S14D.†

In the experiment,^38^ the actin partitioning needed tens of minutes to reach an equilibrium. Thus, to better sample the actin partitioning behaviour, we performed MD simulations of the self-assembly of polypeptides in the presence of one globular actin (G-actin). 50, 100, and 158 pairs of polypeptides, composed of coil Lys/Glu, β sheet Lys/Glu, and coil Lys/Asp, were placed uniformly randomly in the simulations. An example of a self-assembled final state, which was composed of one G-actin and 100 pairs of coil Lys/Asp polypeptides is illustrated in Fig. 3C. The peptides aggregated and the G-actin attached to the coacervate surface. A similar situation also happened for actins with coil Lys/Glu and β sheet Lys/Glu systems. To quantify the partitioning level of actin into the coacervate phase, we computed the actin contact fraction (Fig. 3D). The location of the first valley of RDF (0.6 nm) was used as a cut-off in the contact fraction calculations (Fig. S14C†). We obtained a contact fraction of 0.905 ± 0.011 when the G-actin was embedded into the coacervate phase. Thus, a contact fraction of 0.905 ± 0.011 means actin is fully engulfed by the coacervate, and a contact fraction of 0 means actin stays in the bulk water. Any number in between means that actin is partially wrapped by polypeptide chains. As shown in Fig. 3D, a contact fraction of 0.55–0.7 was observed for all the systems, suggesting that the actin was only partially engulfed by the coacervate phase. In addition, the actin/coacervate contact fraction was slightly higher for coil Lys/Asp than the other two systems. Therefore, we found that both G-actin and F-actin prefer to locate at the surfaces of the polypeptide coacervates and the contact fraction was found to be higher for coil Lys/Asp than Lys/Glu systems.

To further understand how the actin partitions into the coacervate droplet, we simulated well phase-separated coacervate and water slabs at 0.15 M NaCl. The coacervates were composed of 392 pairs of coil Lys/Glu, β sheet Lys/Glu, and coil Lys/Asp polypeptides. A G-actin was placed at the center of the bulk water region. In all the simulations, as expected, the actin diffused to the surface of the coacervate and stayed there for tens of microseconds. An example of G-actin attached to the Lys/Asp surface is illustrated in Fig. 3E. Similar to the previous results, the G-actin prefers to locate at or close to the coacervate phase from the water region. With brute-force MD simulations, one would not be able to distinguish whether the actin preferred location at either the surface or in the bulk region of the coacervate because of the long time scale compared to the simulation time. Thus, we applied umbrella sampling,^39^ an enhanced sampling scheme, to compute the free energy difference of actin partitioning into the coacervate slab.

The histograms from umbrella sampling had good overlaps between neighbouring windows as shown in Fig. S15A–C,† suggesting sufficient sampling. As shown in Fig. 3F, for all systems, the free energy plateaued by a distance of 17.5 nm, which put the actin in the bulk water region. The PMF decreased and reached the minimum when actin moved to a distance of about 10 nm, where actin was close to the coacervate interface. The free energy increased again when actin moved into the coacervate region. Therefore, the coacervate/water interface, where the free energy minimum is located, is the preferred location of the actin in the system. This observation is consistent with the experimental conclusion that actin filaments localize predominantly at the coacervate periphery.^38^ The PMFs of Lys/Glu systems were similar and the depth was deeper than that of the Asp/Lys system, suggesting that actin prefers the interface region more in Lys/Glu coacervate than in the Asp/Lys system.

## Conclusion

We successfully simulated Asp/Lys and Glu/Lys complex coacervates using the Martini 3.0 force field and investigated how the NaCl concentration and pH regulate coacervate formation. We also present a proof-of-concept simulation demonstrating that a complex coacervate composed of different polypeptide chains or stereoisomers can be used as a model bioreactor to regulate the partition or bio-function of the cytoskeletal protein actin. We reconfirmed that the Martini 3.0 model can reproduce the experimental salt concentration dependence^31^ and confirmed the pH dependence of different polypeptide coacervates. We showed that coacervates inside a vesicle can influence the vesicle shape. We also observed that actin partitions spontaneously to the periphery of the coacervate phase, even though different polypeptide pairs correspond to different relative free energy changes between the coacervate bulk and the interface.

## Methods

### Simulation details

We carried out all the simulations using the GROMACS software^40^ and the coarse-grained simulations were conducted with the Martini 3.0 force field.^2,41^ A time step of 20 fs was used with constant NPT (number, pressure, and temperature) dynamics. The Lennard-Jones and Coulomb potentials were shifted to zero at a cut-off of 1.1 nm and the long-range electrostatics was treated using a reaction field (*ε*_rf_ = 15 beyond the cut-off). The neighbor list was updated with the Verlet neighbor search algorithm.^42^ The pressure was coupled with the Parrinello–Rahman algorithm^43^ at 1 bar with a compressibility of 3 × 10^−4^ bar^−1^ and a time constant of 12 ps. The temperatures of the systems were coupled at 298 K using a v-rescale thermostat^44^ with a time constant of 1 ps. The salt concentration was 0.15 M unless otherwise stated.

30-mer Lys, Asp, or Glu polypeptides were generated using the martinize2.py script, downloaded from the GitHub website.^45^ The backbones of polypeptides were built with predefined secondary structures extracted from either all-atom simulations or experimental data. In the case of beta-strands, harmonic (“elastic”) bonds between (1,3) and (1,4) backbone beads with a force constant of 1250 kJ mol^−1^ nm^−2^ were added. The coil Lys/Glu and the β sheet Lys/Glu were the same apart from their secondary structures, meaning that the differences only lay in the bond parameters of the two polymer chains.

The structure of the monomeric G-actin containing ATP was adapted using the crystal structure from the Protein Data Bank (PDB) (ID code 1nwk).^46^ The DSSP^47^ was used to predict the secondary structure elements of each G-actin. The protein was completed by reconstructing the missing terminal residues (residues 1 to 5 and 372 to 375) as random coils and transferring the coordinates of the DNase-binding loop (residues 40 to 57) that were not present in the 1nwk structure from the structure of the actin–DNase I complex (PDB ID code 1atn).^48^ The bond parameters of ATP, the linkage of ATP and Mg to the G-actin, and the way to build F-actin were extracted from ref. 49 (Martini 2 model). The Martini 3.0 bead types used in the ATP model are illustrated in Fig. S13F.† Since ATP was deeply embedded in the actin proteins, it had no contact with the polypeptides as shown in Fig. S13E.† Therefore, because the F-actin model was stable in the simulation, no further validation of the ATP model was needed. Therefore, the model was there only to keep the overall structure of actin stable. The validation of the Martini 3.0 F-actin model is now included in the ESI.† An elastic network with a force constant of *k* = 500 kJ mol^−1^ nm^−2^ was applied in actins between non-linked backbone beads within a cutoff of 0.7 nm. A selection of the GROMACS input parameters and topology files of Martini models are available in ref. 50. Furthermore, a zip file containing example GROMACS simulation setups from Fig. 1–3 is also available in the ESI.†

For the self-assembled salt-dependent simulations, 100 pairs of Asp/Lys or Glu/Asp peptides were randomly dispersed in a 30 × 30 × 30 nm^3^ simulation box (Fig. 1). The peptide to water weight ratio was about 0.618, which is the same as the value used in ref. 31. Even though the peptide density was much higher than the density in the bulk solution, our simulation represented the local peptide structures (in the tens of nanometer size scale) after peptide aggregation. In vesicle pH-sensitive simulations (Fig. 2), we first built coarse-grain (CG) vesicles using the CHARMM-GUI Martini maker^51^ using the Martini 3.0 input topology. The number of lipids within each leaflet of the vesicle was estimated based on the area per lipid. Already phase-separated Asp/Lys or Glu/Asp polypeptides (from the previous simulation) were added to the vesicle and the overlapped water molecules were removed. During equilibration, six artificial pores in the vesicle were kept open with constraints to enhance lipid flip-flop and to equilibrate the numbers of water inside and outside the vesicle.^52^ The vesicle remained spherical during equilibration (with six water pore restraints). The lipid was considered outside the vesicle, when the distance between the center of mass of the vesicle and the NC3 bead was larger than the vesicle radius, which was defined by the mean distance between NC3 beads in lipids of both leaflets and the center of mass of the vesicle. Water was considered inside the vesicle when the distance between water and the vesicle center of mass was shorter than the vesicle radius. The lipid number in the outer leaflet and water bead numbers inside vesicle plateaued (shown in Fig. S20†), suggesting that the vesicle reached an equilibrium. After equilibration, the vesicle was closed by healing the six water pores by switching off the constraints on the lipids. In the actin self-assembly simulations (*e.g.*Fig. 3C), 100 pairs of Asp/Lys or Glu/Asp peptides and a G-actin were uniformly and randomly placed in the simulation box with the polypeptides. At pH = 11, some water near polypeptide residues were replaced by sodium CG beads to ensure the neutrality of the polypeptides within the vesicle of the simulation. In F-actin simulations, an already phase-separated coacervate was placed together with the F-actin (composed of 16 G-actin) in a periodic box of 30 × 30 × 71.5 nm^3^ (Fig. 3A).

For all the systems, simulations were equilibrated for ∼50 nanoseconds using a 10 fs time step. In the production run, a 20 fs time step is applied and the self-assembly process of Glu/Asp polypeptides required 1 to 2 μs to form stable coacervates.^31^ Thus, all the simulations in our study lasted for about 4–5 μs unless otherwise stated.

An all-atomistic model of one pair of Asp and Lys polypeptides solvated in water was carried out using the CHARMM force field.^53^ Both polypeptides adapted l-homochiral structures, which is the most common stereoisomer in nature. The Asp and Lys polypeptides were initially equilibrated in a random coil conformation and then allowed to relax for 600 ns. The equilibration process followed the suggested scheme proposed in CHARMM-GUI.^54^

### Analysis details

The RDFs, partial density, cluster size, root mean square deviation (RMSD), root mean square fluctuation (RMSF) and contact number were calculated using the GROMACS tool gmx rdf, gmx density, gmx clustsize, gmx rms, gmx rmsf and gmx mindist, respectively.^40^ RDF average distances from any CG polymer beads to other molecule beads over the last 500 ns of the trajectory. The locations of the first valley in RDFs are used as a cutoff to compute the cluster size and the contact number. The density profile was normalized by the maximum density of each component. The actin contact fraction was defined as:

1 *f*_actin_ = *c*_actin-peptide_/(*c*_actin-peptide_ + *c*_actin-solvent_)

where the *c* variables represent the numbers of contacts between the CG beads in the two molecule types. *f*_actin_ = 0.905 ± 0.011 when the actin is fully engulfed by the polypeptides, whereas *f*_actin_ = 0 when actin is located at the bulk of the water. Any number in between implies actin is partially wrapped by polypeptide chains.

Sphericity^55^ is a measure of how closely the shape of a vesicle resembles that of a perfect sphere and is defined as:2
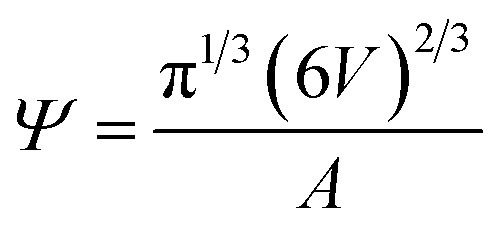
where *V* and *A* are the volume and the surface area of the vesicle. It has been reported that the magnitude of the unidirectional flux is 1 CG water (4 water molecules) per 100 ns through a Martini membrane.^47^ The number of water beads inside the vesicle and its volume barely changed throughout the simulation. Thus, π^1/3^(6*V*)^2/3^ in eqn (2) is basically a constant and we may consider only the 1/*A* part to quantify the sphericity. A higher value suggests the vesicle better resembles a perfect sphere.

The membrane thickness, area per lipid and surface area were computed with the Fatslim software.^56^ The second rank order parameters were calculated according to3
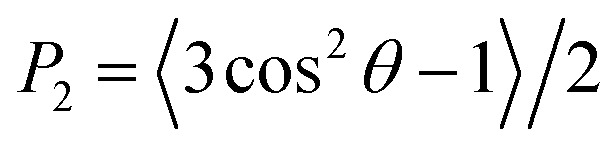
*θ* is the acute angle between the CG bond and the line connecting the center of mass of the vesicle to the center of the CG bond in the vesicle. The mean order parameter is the average value of the order parameters of lipids in the vesicle in a particular frame.

Free energy was calculated by performing umbrella sampling methods.^39^ The center of the mass distance between the G-actin and the coacervate phase was set as the reaction coordinate. A G-actin and 392 pairs of Asp/Lys or Glu/Asp peptides were placed in a simulation box of 22 × 22 × 44 nm^3^. 190 windows separated by 0.1 nm were used with a distance ranging from the center of the coacervate (0 nm) to the coacervate water (∼19 nm). A spring constant of 1500 kJ mol^−1^ nm^−2^ was used for the umbrella sampling potential. The compressibility of the pressure in the *x* and *y* directions was set to 0, but kept the coupling on in the *z*-direction. Each window lasted for 500 ns. The weighted histogram analysis method was used to analyze the umbrella sampling results.

## Data availability

A Zip file of the example input files for the simulation setups in Fig. 1−3 is available as ESI.†

## Author contributions

Y. L. designed the project, performed the research, and analyzed the data. All the authors discussed the results, wrote the manuscript, and revised the final version of the text.

## Conflicts of interest

There are no conflicts to declare.

## Supplementary Material

SC-014-D2SC01164F-s001

SC-014-D2SC01164F-s002
