# Supplementary material for: Capturing coacervate formation and protein partition by molecular dynamics simulation
Source: Chem Sci. 2022 Dec 24;14(5):1168–75. doi: 10.1039/d2sc01164f (PMC9891350; doi:10.1039/d2sc01164f)
Supplement: SC-014-D2SC01164F-s001 [file SC-014-D2SC01164F-s001.pdf]

Electronic Supplementary Material (ESI) for Chemical Science.
This journal is © The Royal Society of Chemistry 2022

Capturing coacervate formation and protein partition by molecular dynamics simulation

Yang Liu,[a,b] Xinyan Wang,[b] Zhili Wan,[c] and To Ngai*[b] and Ying-Lung Steve Tse*[b]

[a] College of Polymer Science and Engineering, State Key Laboratory of Polymer Materials Engineering, Sichuan University, Chengdu 610065, China

[b] Department of Chemistry, The Chinese University of Hong Kong, Hong Kong, China

[c] School of Food Science and Engineering, South China University of Technology, Guangzhou, China

*Corresponding authors

E-mail: tongai@cuhk.edu.hk(T. Ngai), stevetse@cuhk.edu.hk (Y.-L.S. Tse)

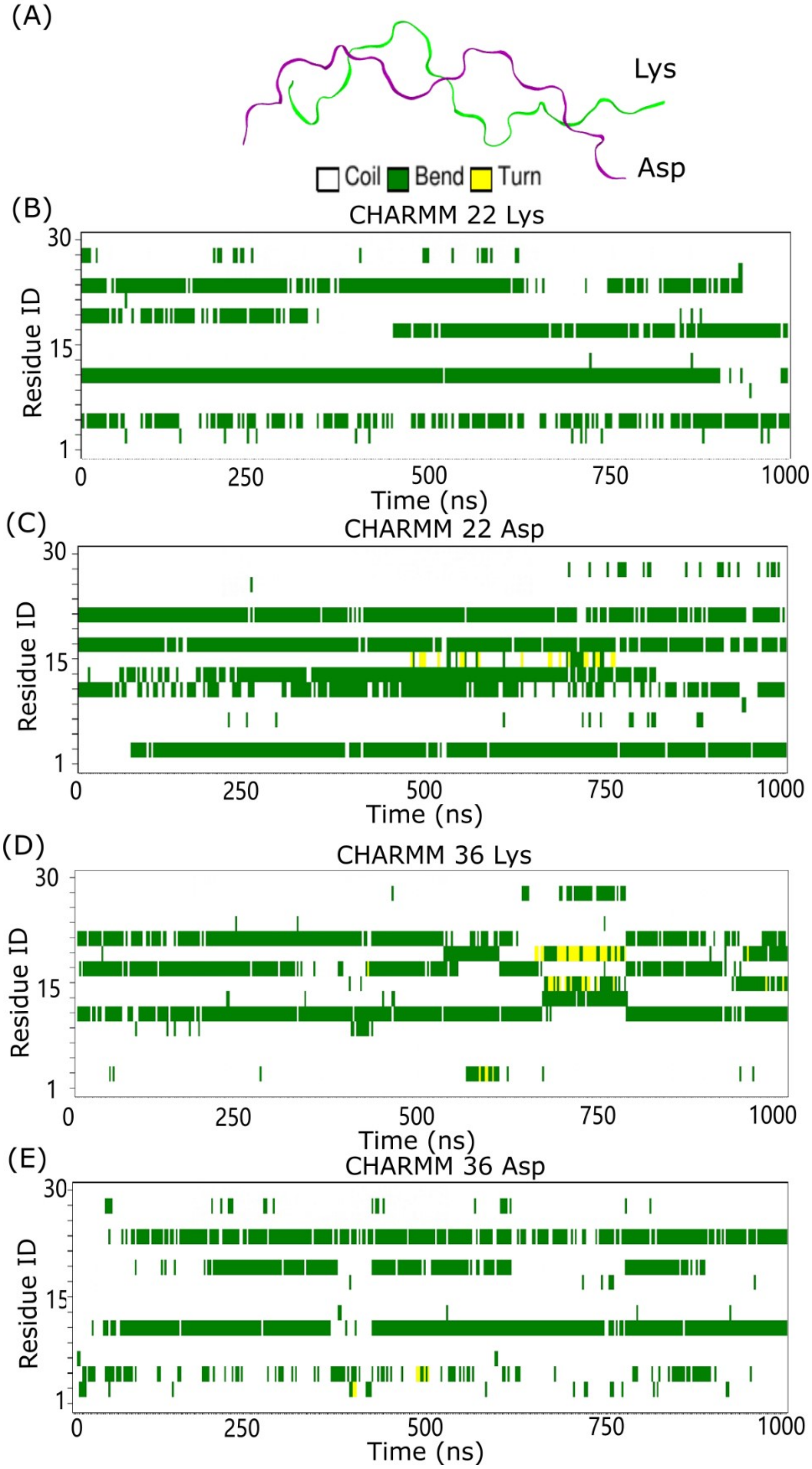

Fig. S1 Visualization and secondary structures of one pair of Asp30 and Lys30 peptides, which were initially equilibrated in a random coil conformation and then allowed to relax for 1000 ns. Both polypeptides were L-homochiral, which is the most common stereoisomer. (A) illustrates the end state of the simulation. Water and ions are not shown. (B-E) are maps of the secondary structures for Lys30 and Asp30 simulated with the different versions of CHARMM force field as a function of time.

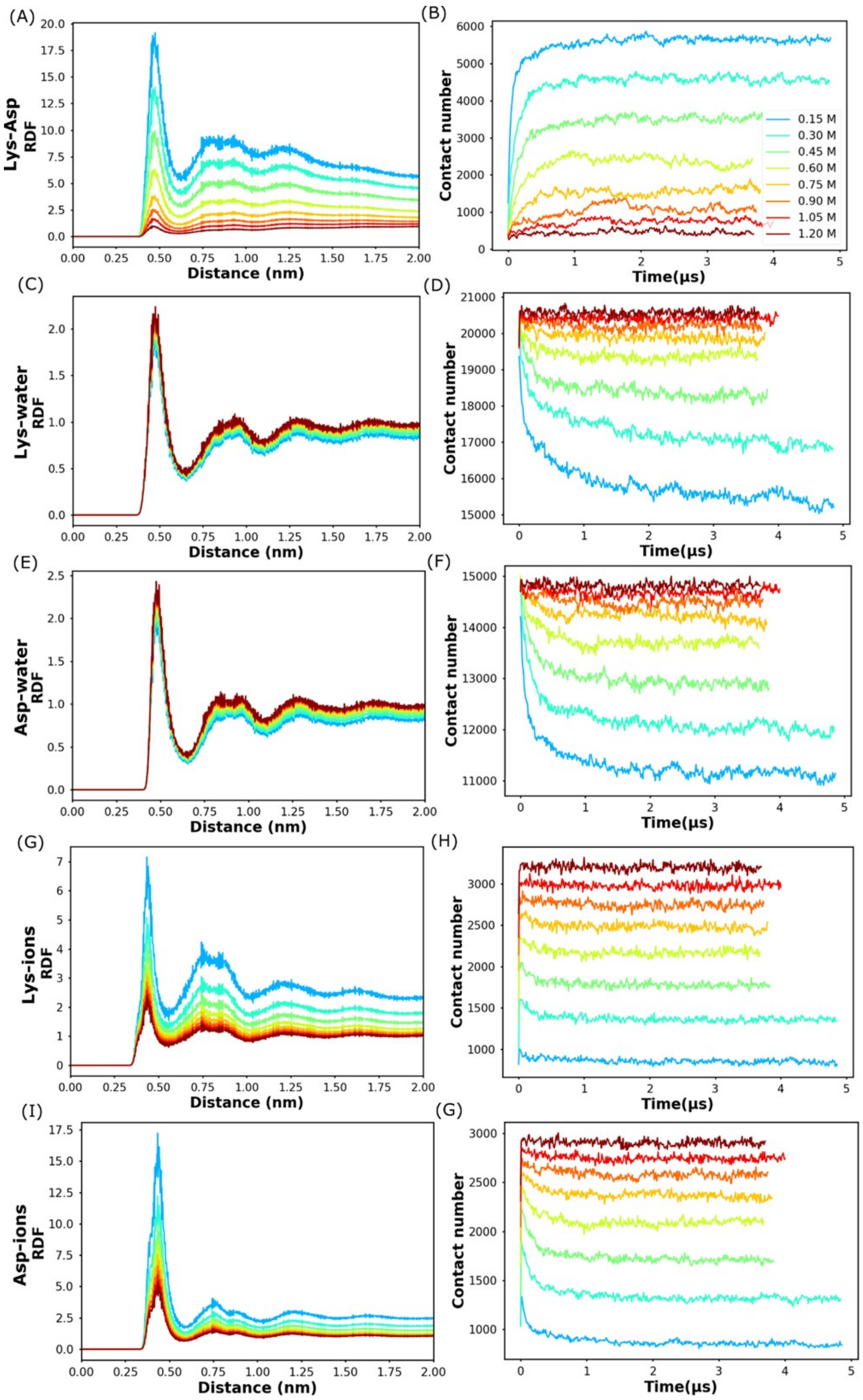

Fig. S2 Quantification of the salt concentration effect on Lys/Asp coacervate. The first column shows the Radial Distribution Function (RDF) and the second column shows the contact number between components. A cut-off of 0.6 nm was the largest distance to be considered as contact.

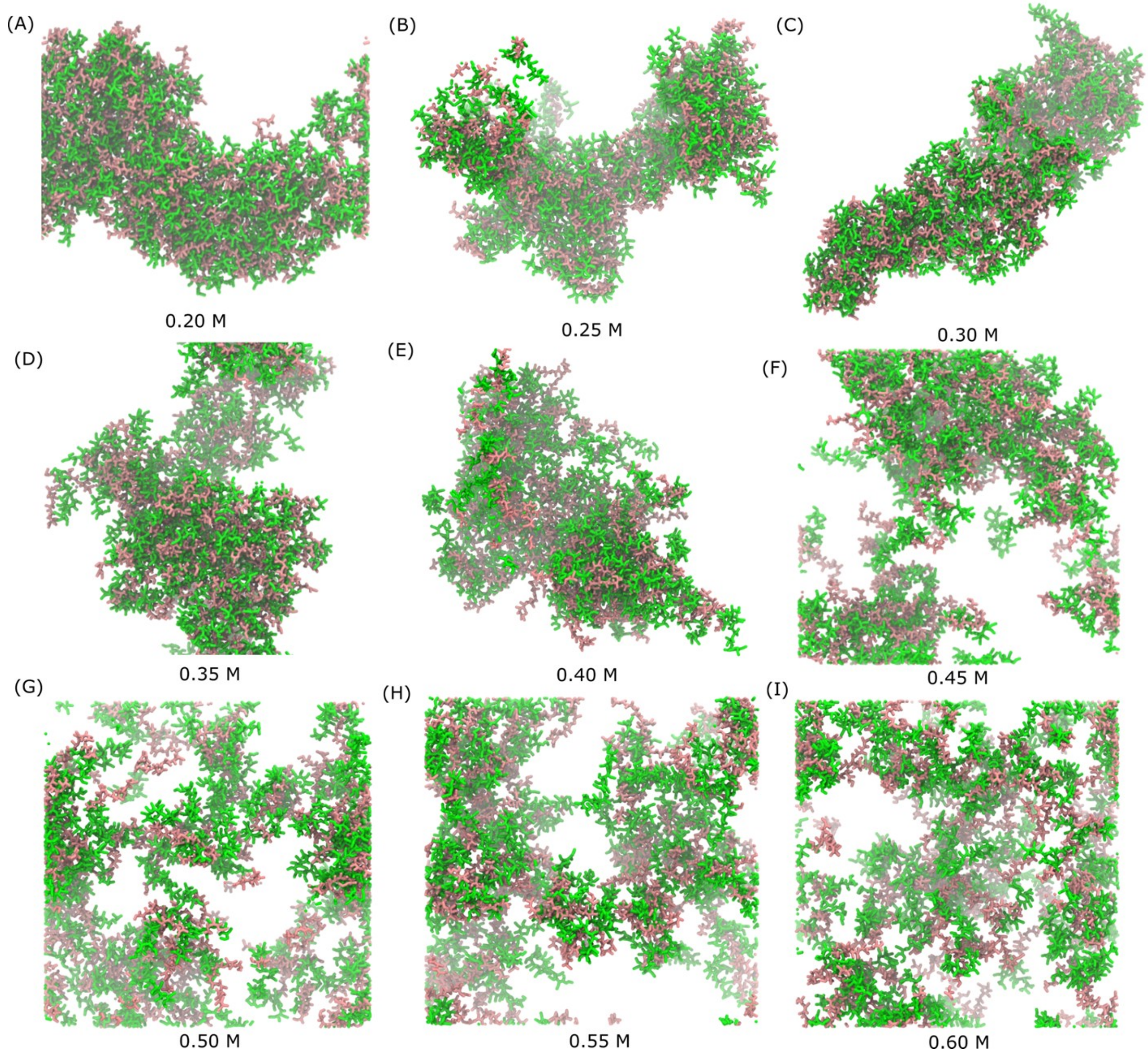

Fig. S3 Salt-dependence of Lys/Glu (coil secondary structure) coacervate. (A-F) Snapshots of the final state of 100 Lys30 and 100 Glu30 polymers systems at different salt concentrations. Lys30 and Glu30 are shown in green and pink, respectively. Water and ions are not shown.

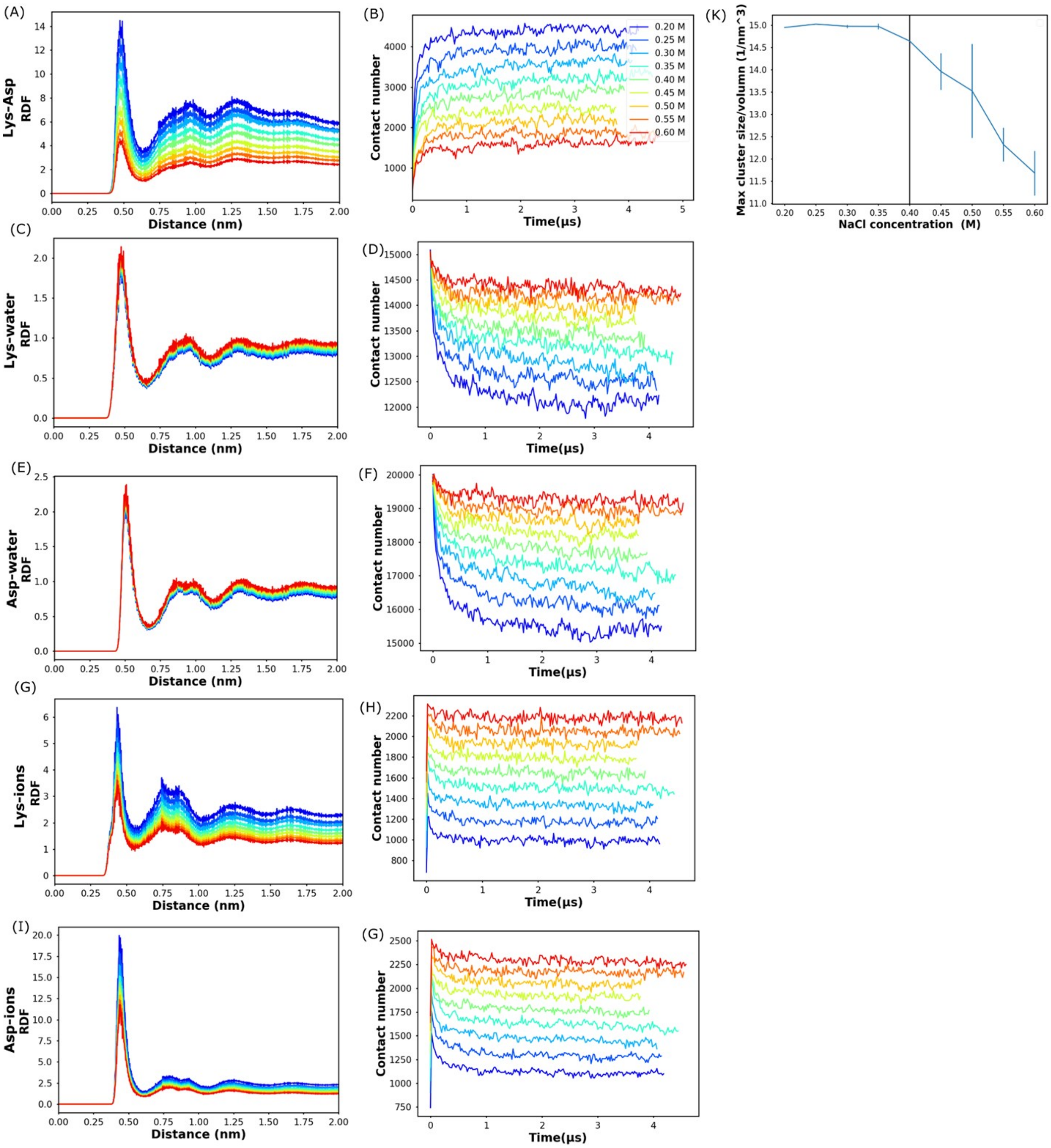

Fig. S4 Quantification of the salt concentration effect on Lys/Glu (coil secondary structure). The first column shows the Radial Distribution Function (RDF) and the second column shows the contact number between components. A cut-off of 0.6 nm was the largest distance to be considered as contact. (K) Maximum peptide cluster size at different salt concentrations normalized by the volume of each system. The vertical line represents the experimental transition salt concentration.[1]

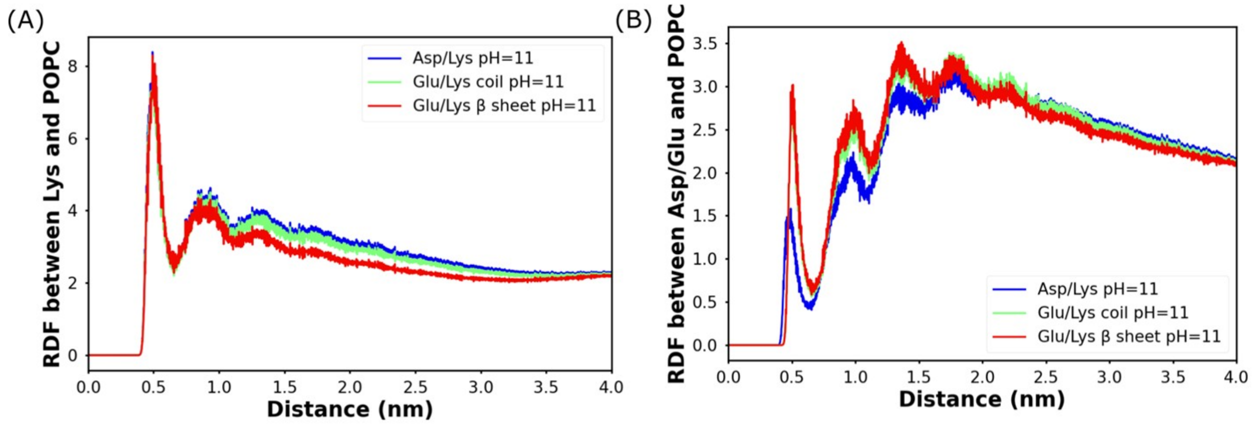

Fig. S5 Radial Distribution Function (RDF) of the coacervate vesicle system. (A) and (B) show the RDF between polypeptides and POPC lipids, in which only the heads of the POPC (PO_4 and NC_4 beads) were used as reference points in the calculations.

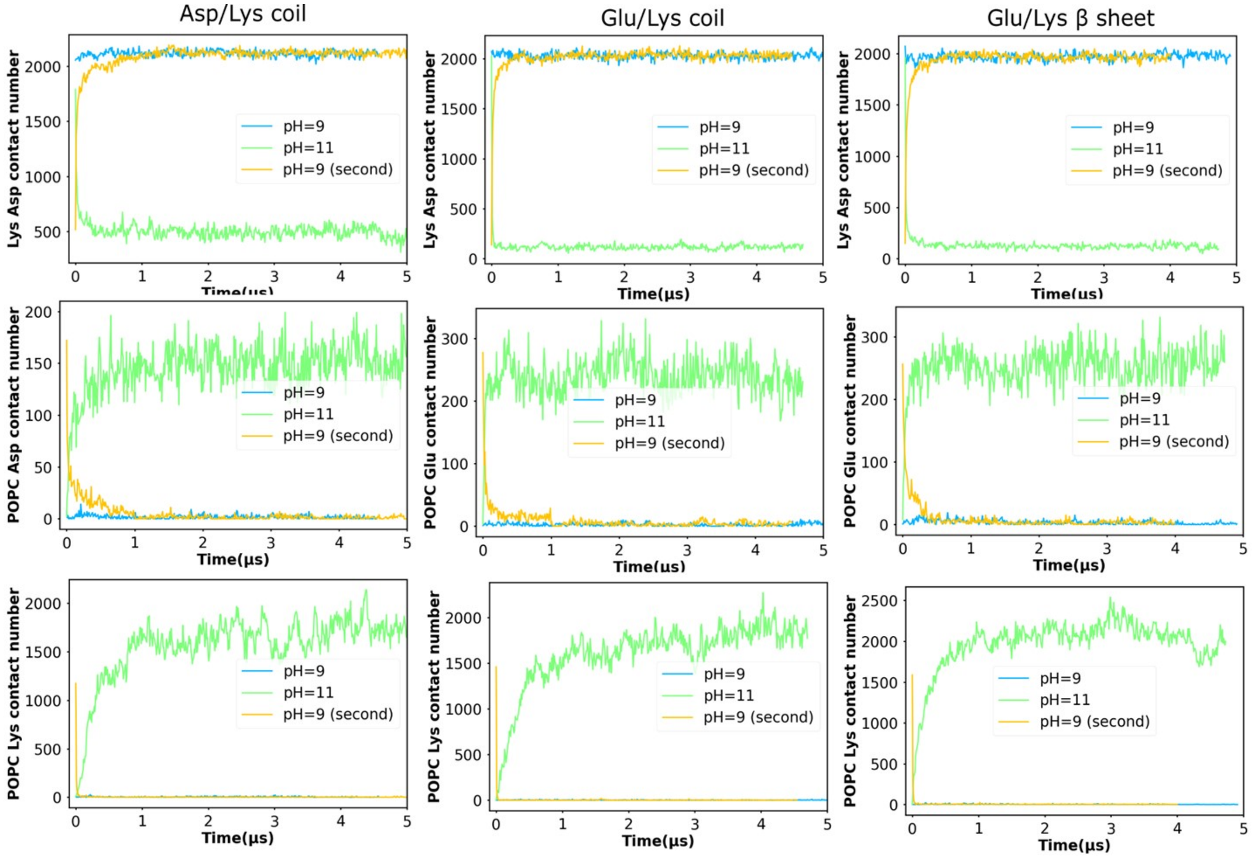

Fig. S6 Contact number for coacervate vesicle system systems at different pH values. The cut-off to be considered as contact was the location of the first valley of RDF (first column of Fig. S4 and Fig. S5) between the two target molecules.

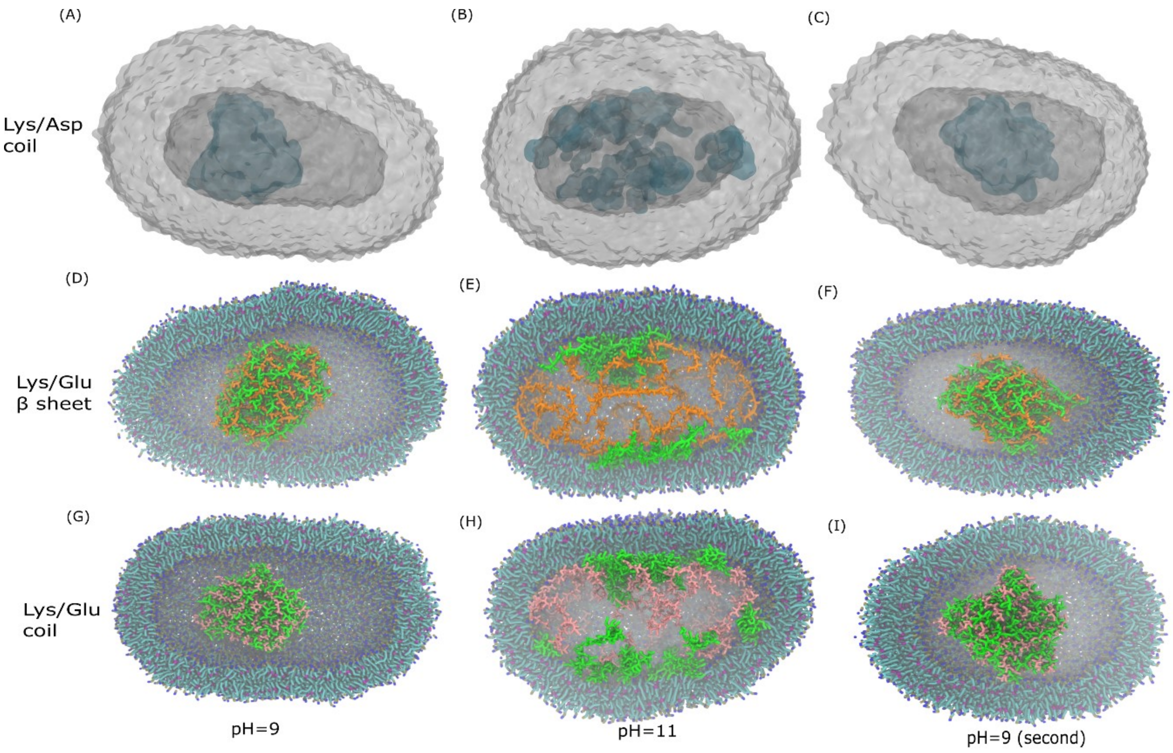

Fig. S7. Snapshots of the final state of polypeptides and vesicle systems at different pH values. The Lys/Asp coacervate and vesicle are represented in blue and white, respectively.

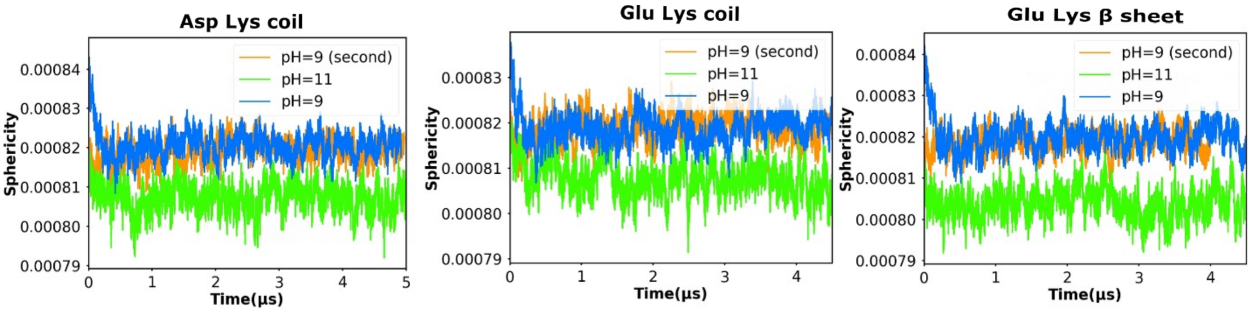

Fig. S8. Sphericity of coacervate vesicle systems at different pH values as a function of time.

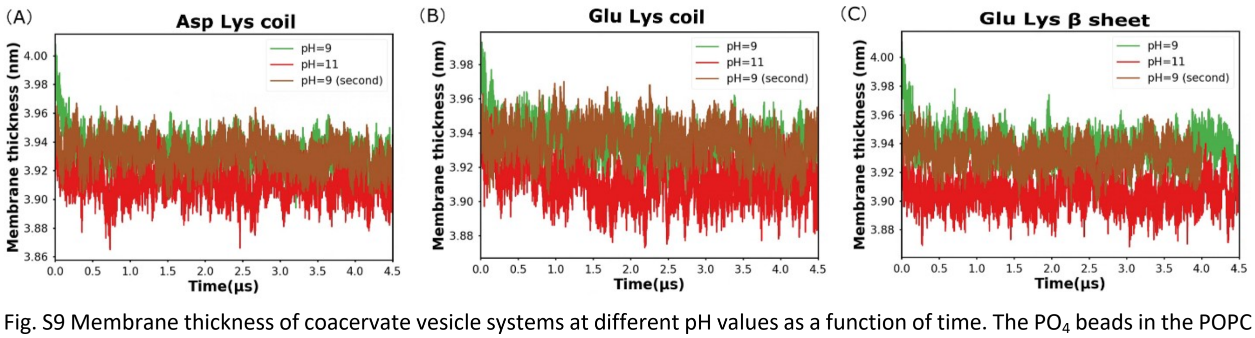

Fig. S9 Membrane thickness of coacervate vesicle systems at different pH values as a function of time. The PO_4 beads in the POPC lipids were used as reference to compute the membrane thickness.

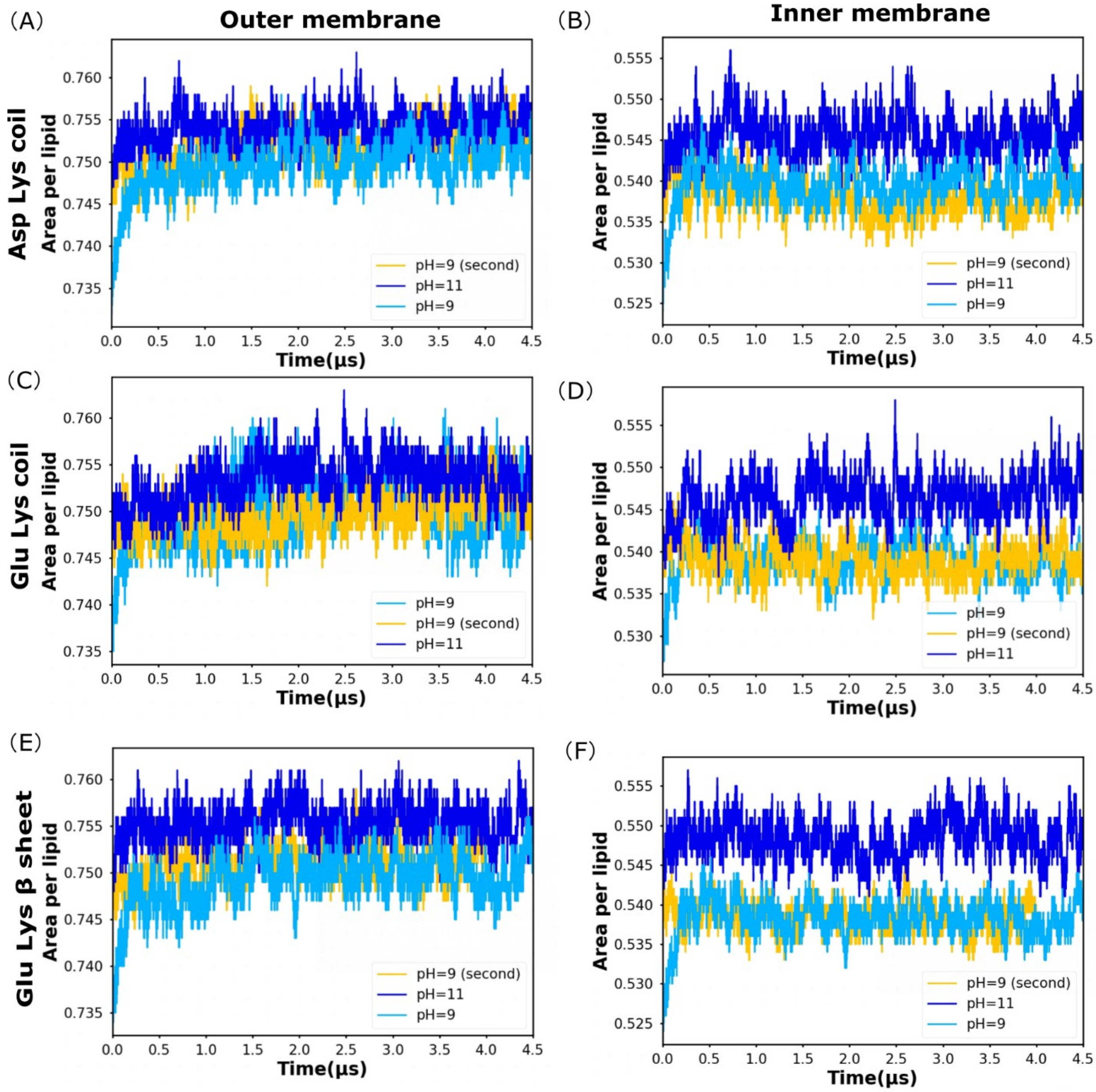

Fig. S10 Area per lipid of coacervate vesicle system systems at different pH values as a function of time. Inner and outer leaflets were considered separately.

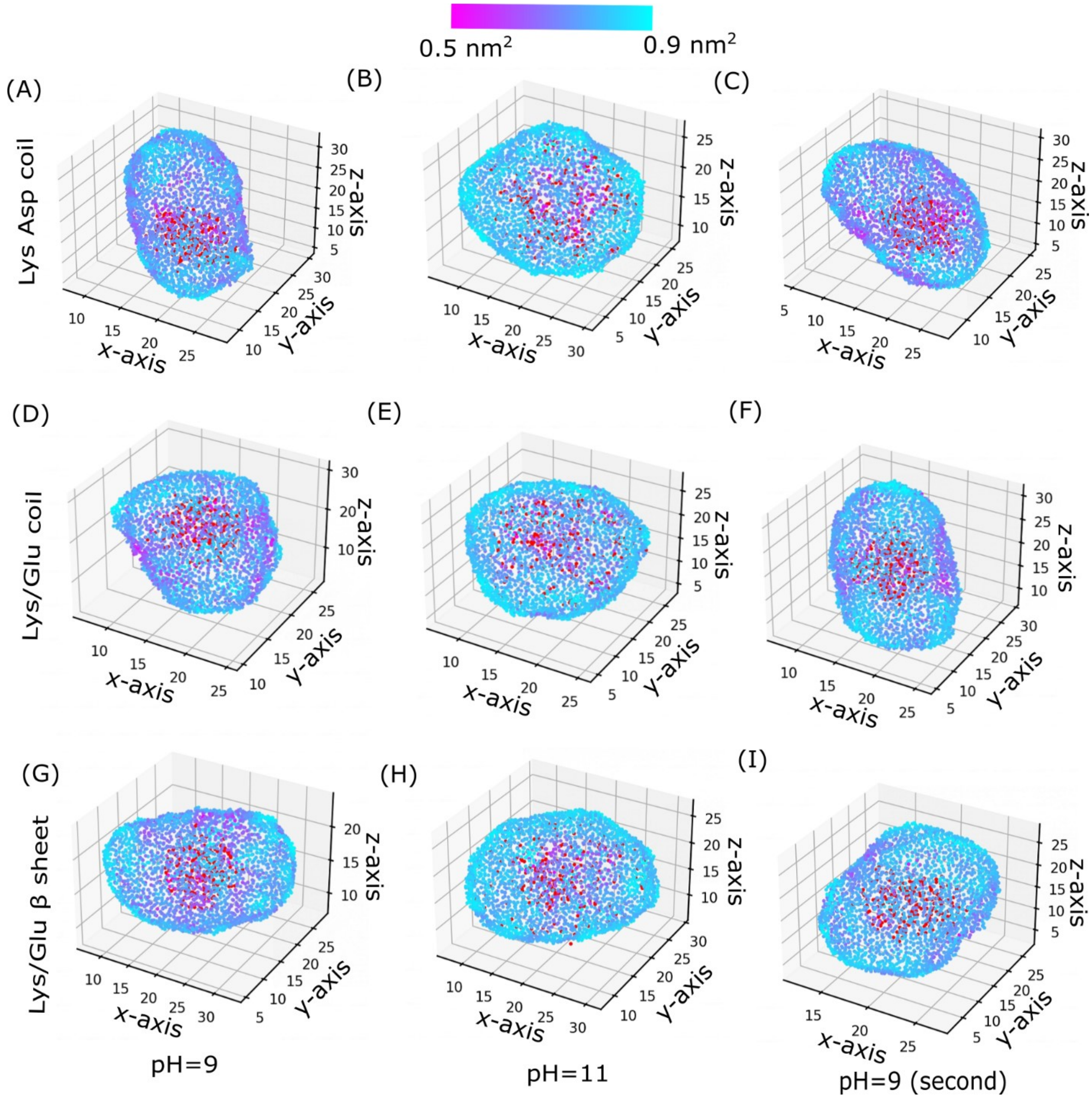

Fig. S11 Area per lipid of inner leaflets of coacervate vesicle systems at different pH values. The units on x, y and z axes are *nm*.

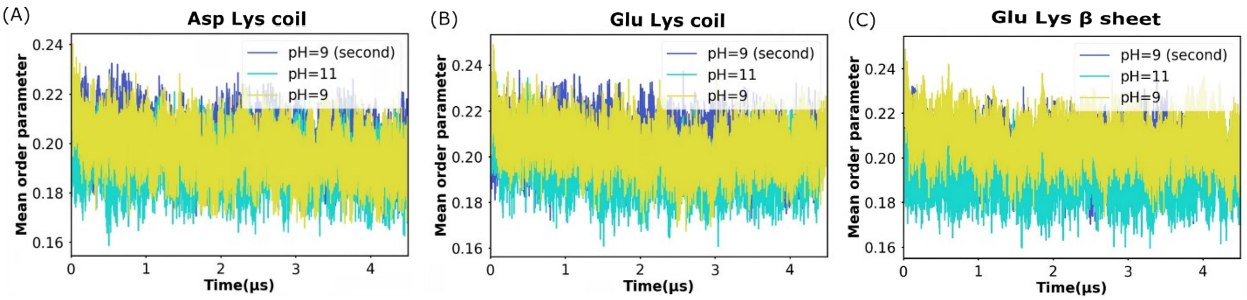

Fig. S12 Mean order parameter of lipids in coacervate vesicle systems at different pH values as a function of time.

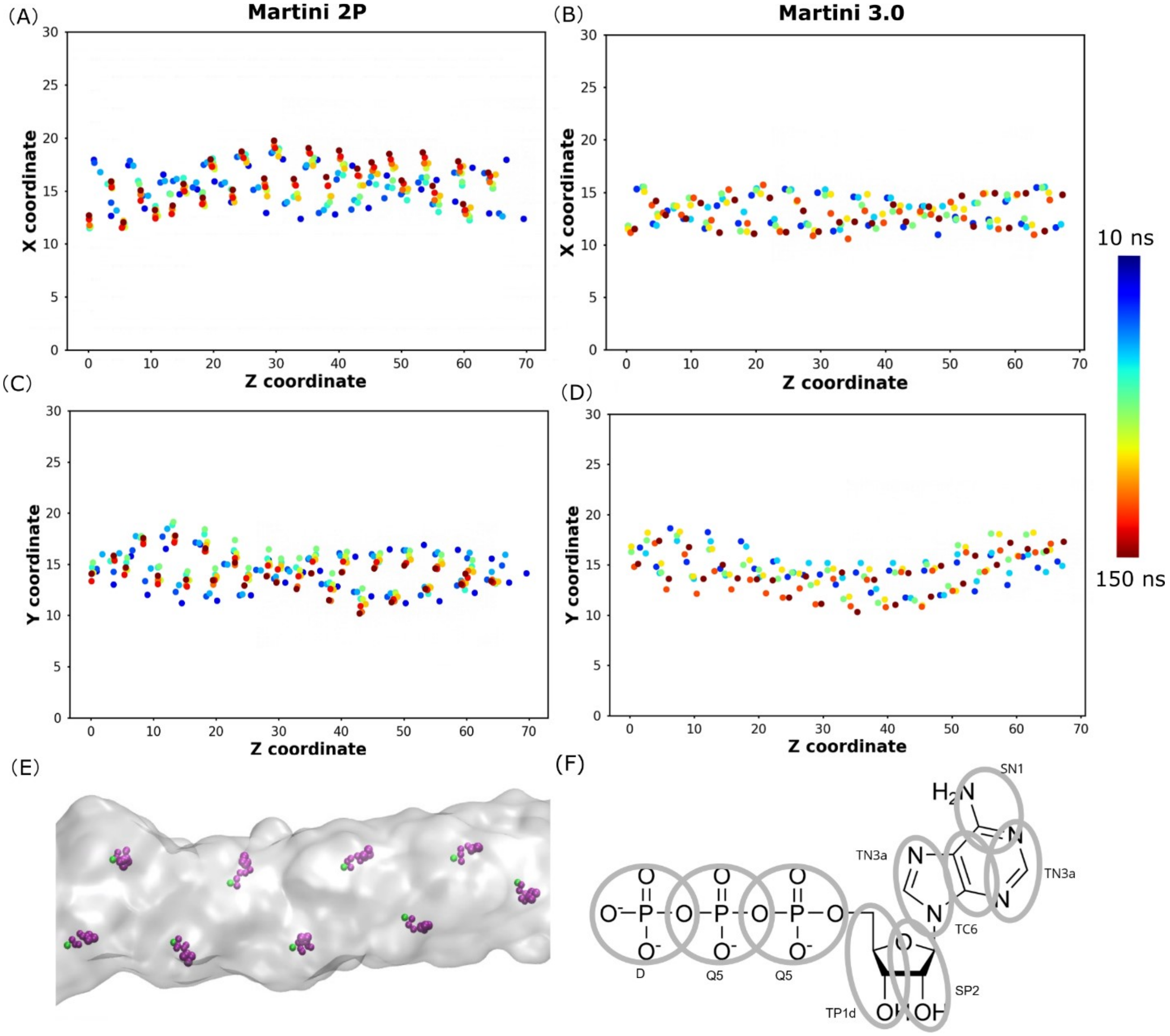

Fig. S13 Comparison between Martini 2P and Martini 3 actin model. (A)-(D) show the coordinates of Mg beads embedded in each actin monomer. The centers of mass of the proteins were fixed for the analysis. The unit was nm for the X, Y, and Z axes. (E) show the snapshot of the final state of Martini 3 actin model. Actin is shown as the transparent surface. Mg and ATP beads are colored by green and purple. (F) shows the chosen bead types for Martini 3.0 ATP model.

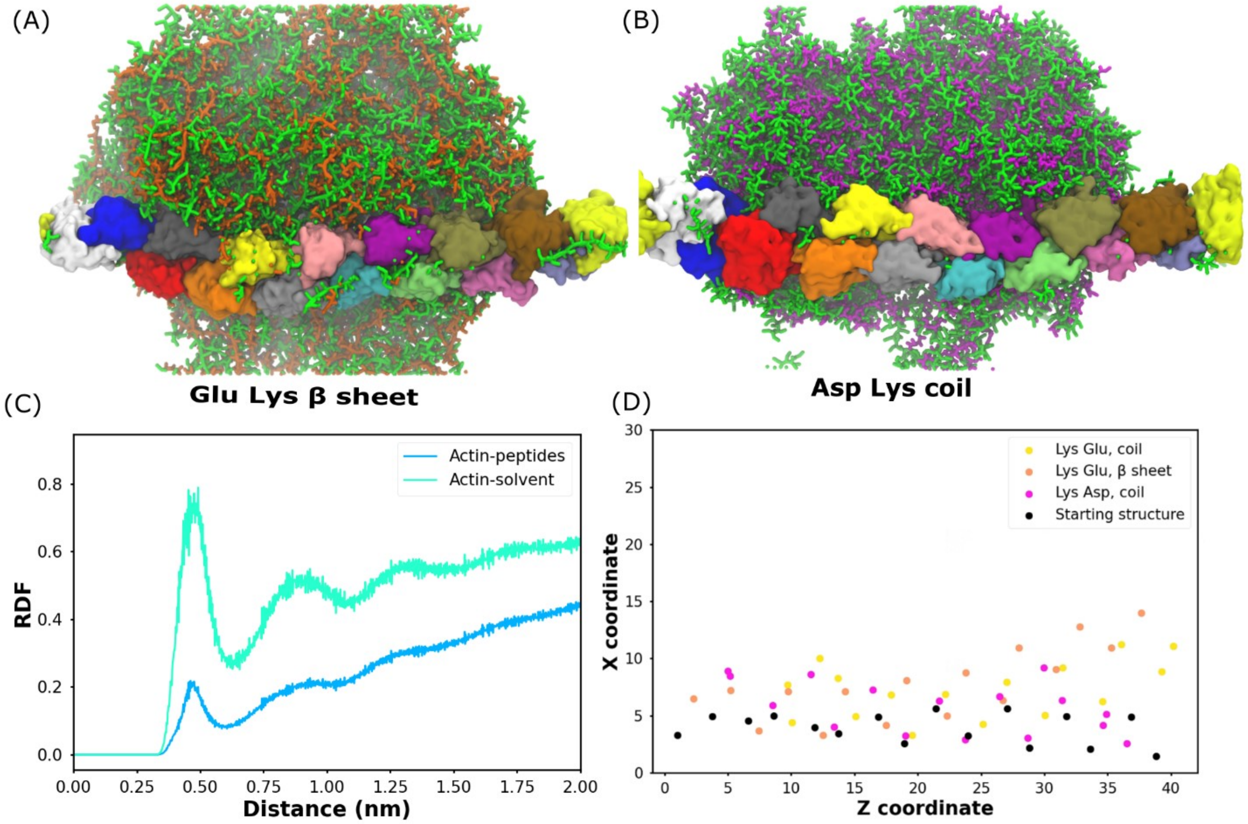

Fig. S14 F-Actin attached to the surface of multi-peptide coacervate formed by (A) Lys/Glu β-sheet and (B) Lys/Asp coil. "Solvent" included both water molecules and salt ions. A cut-off of 0.6 nm was the largest distance to be considered as contact in the actin contact fraction calculations. (C) shows the RDF between the CG beads of the stated molecule types. (D) Initial and final coordinates of Mg CG beads embedded in each actin monomer. The centers of mass of the proteins were fixed for the analysis. The unit was nm for the X and Z axes.

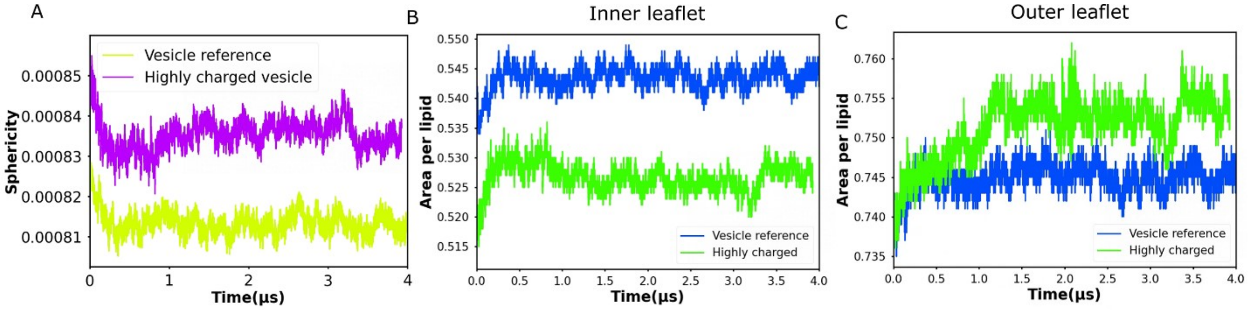

Fig. S15 Osmotic imbalance across the vesicle membrane. The vesicle reference system includes 0.15 M NaCl (aq). Apart from the 0.15 M NaCl (aq), additional 2250 sodium ion and 2250 chloride CG beads were added inside to form the highly charged vesicle.

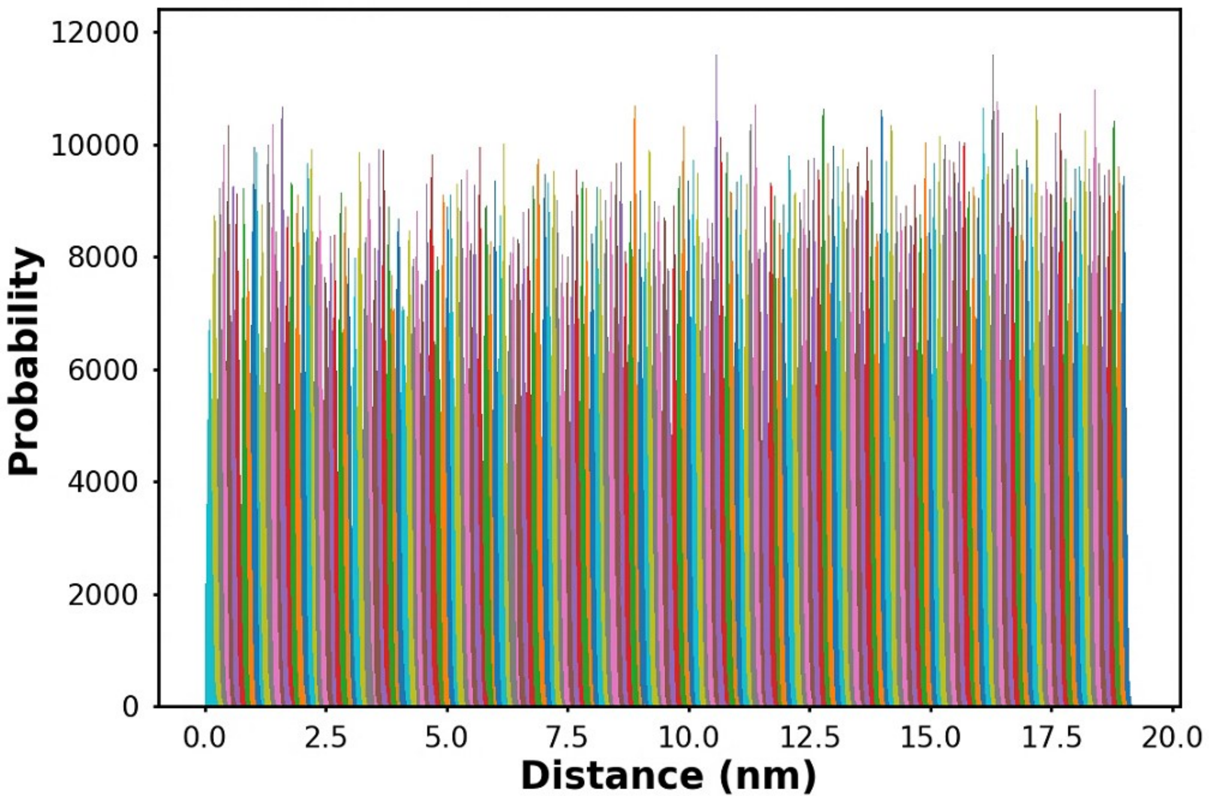

Fig. S16 Histogram of the distances between the centers of mass of G-actin and coacervate in each window in the coil Lys/Asp system.

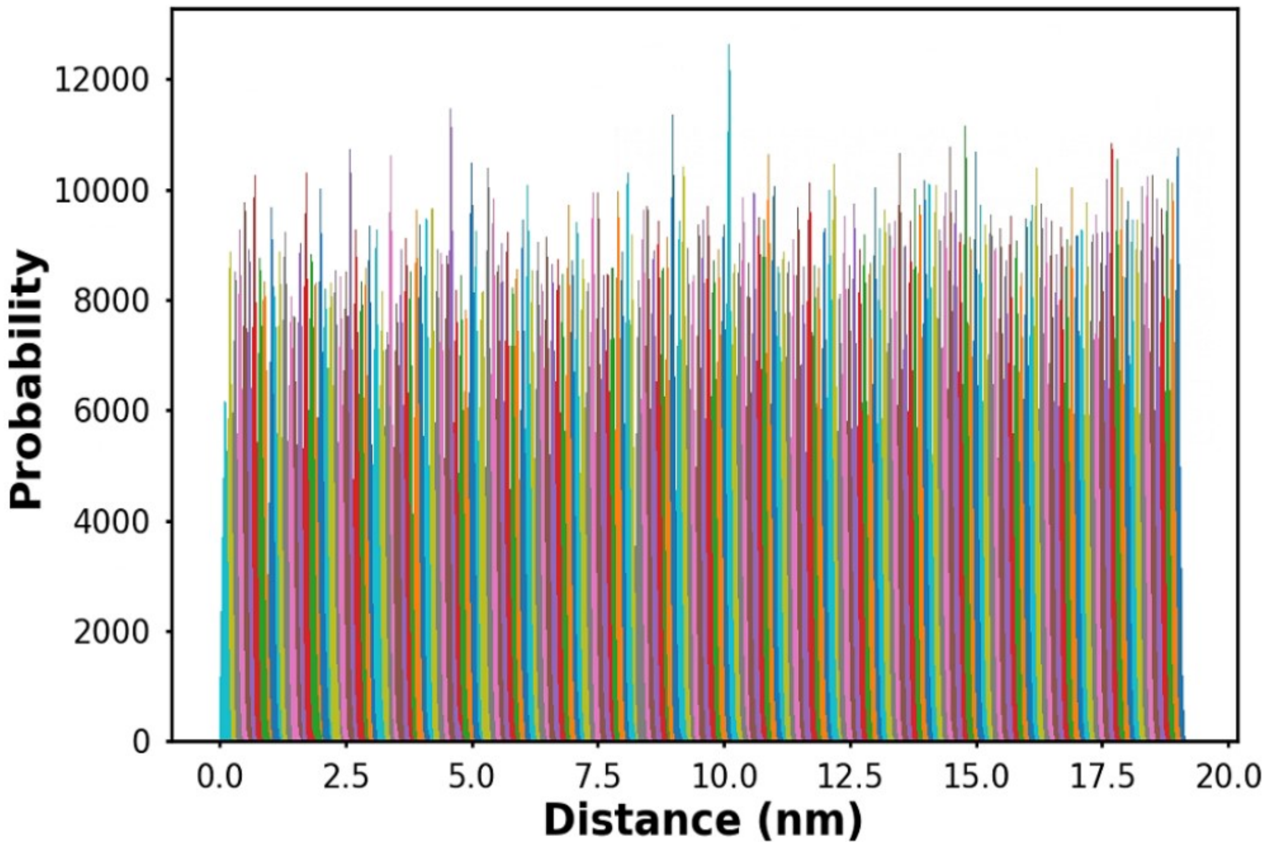

Fig. S17 Histogram of the distances between the centers of mass of G-actin and coacervate in each window in the coil Lys/Glu system.

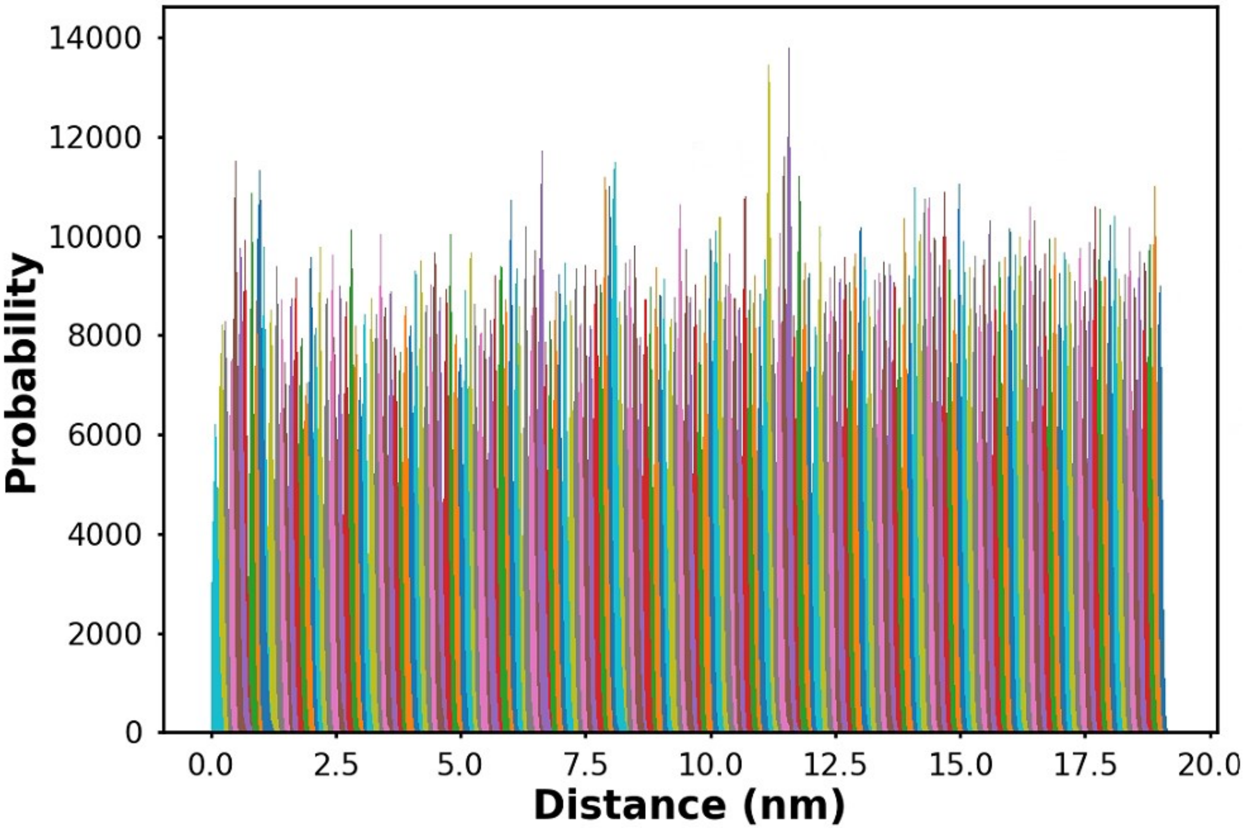

Fig. S18 Histogram of the distances between the centers of mass of G-actin and coacervate in each window in the β sheet Lys/Glu system.

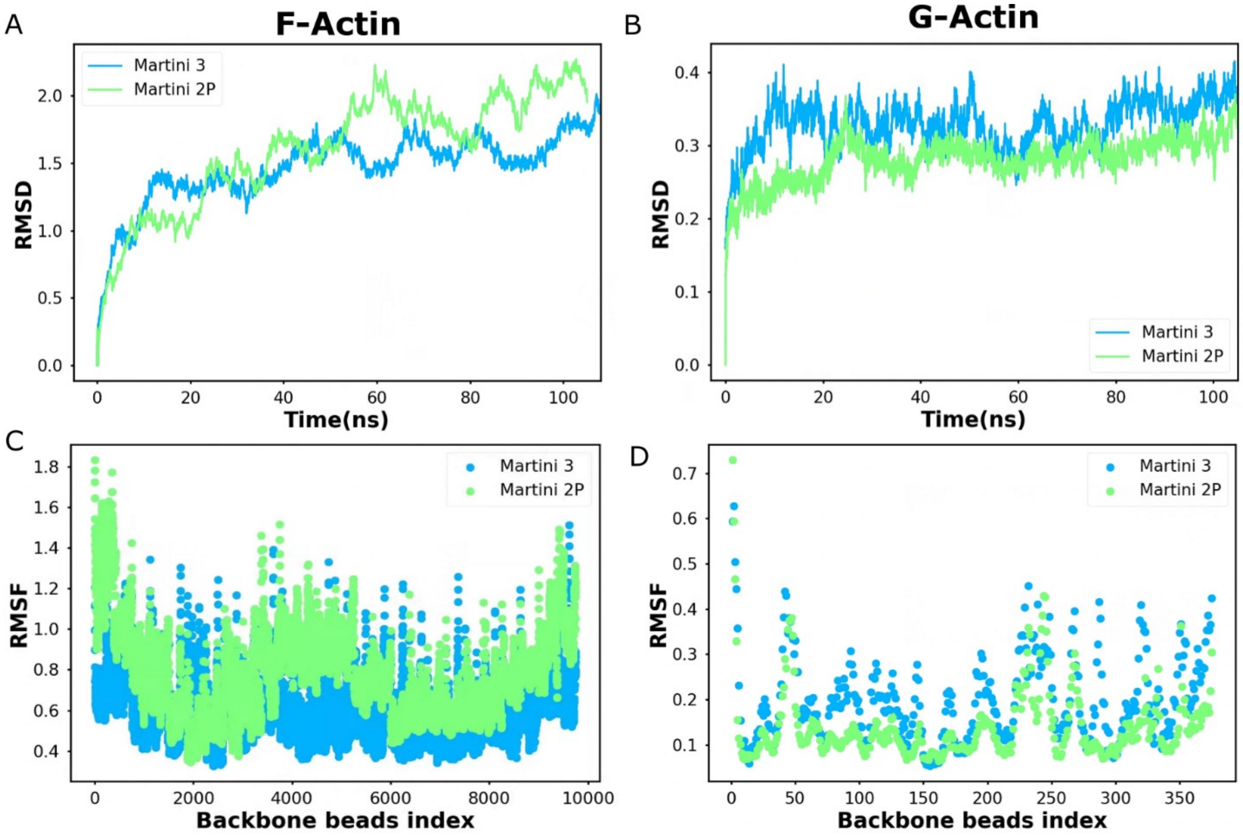

Fig. S19 Comparison between Martini 2P and Martini 3 actin models. (A-B) show the actin backbone root mean square deviation (RMSD), in unit of nm, in relation to the initial structures as functions of time. (C-D) illustrate the actin backbone root mean square fluctuation (RMSF), in unit of nm, of monomers in relation to the average structure. Both RMSD and RMSF are used to quantify the stability of the protein structure.

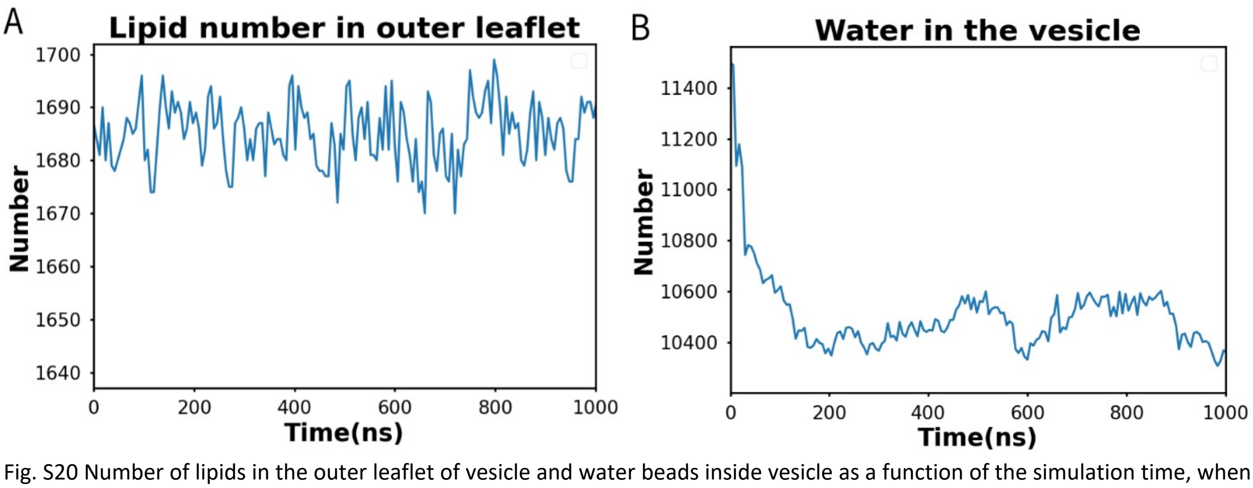

Fig. S20 Number of lipids in the outer leaflet of vesicle and water beads inside vesicle as a function of the simulation time, when the six artificial pores were still open.

Salt concentration effect on Glu/Lys coacervate

We also investigated the salt concentration effect on the coacervate composed of glutamic acid (Glu) and Lys polypeptides. Two common enantiomers of the two peptide polymers are considered: poly-L-lysine and poly-L-glutamic acid, as well as, poly-L-lysine and poly-(D, L)-glutamic acid. In both simulation and experimental findings,[1] β-sheet structures were reported for poly-L-lysine and poly-L-glutamic acid pairs. The β-sheet Glu/Lys (β sheet Lys/Glu) coacervate was previously simulated with Martini 3.0 force field (both beta and final versions) and the transition from coacervation to non-coacervation occurs from 0.36 M to 0.47 M NaCl concentration in the final version.[2] In this paper, we considered the combination of the poly-L-lysine and poly-(D, L)-glutamic acid. Since no stable secondary structures were found for the two polypeptides after they attached to each other,[1] we adopted the coil secondary structure for Glu/Lys polypeptides. We randomized the coil Glu/Lys poly-peptides (Coil Lys/Glu) with a uniform distribution at NaCl concentrations from 0.20 M to 0.6 M. The coacervate is formed at 0.15 M, while gradually dissolving as the salt concentration increases as illustrated in Fig. S3 Similar to the analysis for Asp/Lys systems, the RDF, contact number, and the maximum peptide cluster size was computed for Glu/Lys coacervates as shown in Fig. S4. We found that the transition happens between 0.4 M and 0.55 M NaCl, which agrees with the experimental value of about 0.4 M.[3]

Comparison between Martini 2P and Martini 3.0 actin model

We ran simulations composed of 26 actins-ATP-Mg monomers solvated in water at the salt concentration of 0.15 M NaCl (aq) using both Martini 2P and Martini 3.0. The actins-ATP-Mg monomers were connected to form an F-actin as described in Ref 13. The coordinates of Mg embedded inside each actin monomer were used as a reference (Fig. S13E) to compare the actin structures simulated with the two versions of the Martini models (Fig. S13A -S13D). The Martini 3.0 actin monomers stayed in F-actin structure and the locations of the Mg beads in F-actin using both versions were similar. In addition, both RMSD and RMSF of the two versions of the Martini models are similar, suggesting close stability and flexibility properties of the actin proteins in the two models. Therefore, we believe the Martini 3.0 actin model is reasonable. Noted that we only hoped to investigate the interactions between the actin proteins and polypeptides, and the elastic property of Martini 3.0 F-actin was not validated against experimental data or Martini 2P model.

Osmotic imbalance of vesicle systems

We also investigated the osmotic imbalance across the vesicle membranes. Pure POPC vesicles without multi-peptides were considered here and the membrane properties were quantified in Fig. S15. 0.15 M NaCl (aq) was added to the vesicle reference system. Apart from 0.15 M NaCl (aq), additional 2250 sodium and 2250 chloride CG beads were added into the highly charged vesicle, the ion number of which is equivalent to 30 pairs of muti-peptide chains in Fig. S7. As shown in Fig. S15, the osmotic imbalance of the highly charged vesicle increased the sphericity and decreased the area per lipid of the membrane when compared to the reference system. This trend agrees with the reference vesicle system when compared to the osmotic imbalance vesicle multi-peptide system at pH=9 in Fig. 2F, but with a stronger effect. This is because the multi-peptides in vesicle tend to aggregate in the vesicle and barely contacts with the vesicle inner

surfaces, while the ions in highly charged vesicle tend to diffuse closer to the zwitterionic POPC lipids, leading to stronger effects on vesicle shape.

Reference

1. S. L. Perry, L. Leon, K. Q. Hoffmann, M. J. Kade, D. Priftis, K. A. Black, D. Wong, R. A. Klein, C. F. Pierce, 3rd, K. O. Margossian, J. K. Whitmer, J. Qin, J. J. de Pablo and M. Tirrell, *Nat. Commun.*, 2015, **6**, 6052.
2. M. Tsanai, P. W. J. M. Frederix, C. F. E. Schroer, P. C. T. Souza and S. J. Marrink, *Chem. Sci.*, 2021, **12**, 8521-8530.
3. L. Li, S. Srivastava, M. Andreev, A. B. Marciel, J. J. de Pablo and M. V. Tirrell, *Macromolecules*, 2018, **51**, 2988-2995.
4. C. F. E. Schroer, L. Baldauf, L. van Buren, T. A. Wassenaar, M. N. Melo, G. H. Koenderink and S. J. Marrink, *Proc. Natl. Acad. Sci. U. S. A.*, 2020, **117**, 5861-5872.
